# How Does Energy Intake Change in China? A Life Cycle Perspective

**DOI:** 10.3390/nu16010043

**Published:** 2023-12-21

**Authors:** Guojing Li, Yulin Li, Qiyou Luo, Hongwei Lu, Runqi Lun, Yongfu Chen

**Affiliations:** 1 Institute of Agricultural Resources and Regional Planning, Chinese Academy of Agricultural Sciences, Beijing 100081, China or liguojing@caas.cn (G.L.); luoqiyou@caas.cn (Q.L.); 82101221355@caas.cn (H.L.); 2 State Key Laboratory of Efficient Utilization of Arid and Semi-Arid Arable Land in Northern China, Beijing 100081, China; 3 South Subtropical Crop Research Institute, China Academy of Tropical Agricultural Sciences, Zhanjiang 524091, China; sscri@catas.cn; 4 Agricultural Production and Resource Economics, Technical University of Munich, 85354 Freising, Germany; 5 College of Economics and Management, China Agricultural University, Beijing 100083, China

**Keywords:** Chinese urban residents, energy intake, life cycle, adult equivalent scale

## Abstract

China’s population is showing the characteristics of “fewer children” and “aging”, which will have a long-term and far-reaching impact on the food and nutritional needs of China and the world. In this paper, adult equivalent scale (*AES*) variables representing the household population structure were introduced into the energy intake model to quantify the impact of population structure changes on energy intake and reveal the characteristics of energy intake changes in the life cycle of Chinese residents. It is found that the change in the population structure has a significant impact on the energy intake of urban households in China, and the elasticity is 0.446. The energy intake of Chinese elderly over the age of 60 shows a trend of first increasing and then decreasing, especially after the age of 65, which indicates that the aging will promote a decline in food consumption in China. The energy intake of 1–10-year-old children, 22–30 year old, and 40–45 year old women all showed a change of first a decrease and then an increase, which may be related to parents’ care for young children and women with children’s management of body size. The average household size expressed by the adult equivalent scales of energy consumption is 2.341, which is smaller than the 3.052 calculated by the population number. The conclusion indicates that the prediction of the food and nutritional demand should be adjusted according to the changes and differences in accordance with the intensification of “fewer children” and “aging”, as well as the life cycle changes in residents’ energy intake, which is conducive to the formulation of food and nutrition security policies.

## 1. Introduction

The coexistence of insufficient and excessive nutrient intake has become a major challenge for countries around the world, especially developing countries. For example, in 2021, the number of people in moderate or severe food insecurity reached one-third of the world’s total population, while the proportion of adults in the overweight or obese range reached 40% of the world’s adult population, and the proportion of children reached 20% [1]. Residents of China and India, with their large populations and rapidly growing economies, face a dietary transformation, with overconsumption of refined grains and red meat and underconsumption of vegetables, fruits, and dairy [2]. However, in some African countries with rapid population growth and slow agricultural development, there are problems such as single food types and insufficient energy intake [3]. In the future, the population development and dietary nutritional development of similar countries such as China and India may be similar to that of developed countries such as Japan and South Korea, and whether residents of other developing countries can be guaranteed in the population transition and whether they can learn from the population development transition of China and India have become key topics of research in the world.

Nutrition theory suggests that there are significant differences in the daily energy expenditure of life-sustaining metabolism and physical activity in populations of different ages and genders [4]. Therefore, changes in the size and age structure of family members will lead to changes in the household food consumption and energy intake, which will have an impact on the food and energy consumption of the whole country [5,6]. If the influence of household demographic factors on residents’ energy intake is ignored and, for example, a family consisting of three young people and a family consisting of an elderly person and a child are regarded as households of the same population size, then the analysis of residents’ food demand and energy intake predictions will be biased [7,8]. Therefore, the study of the impact of population structure change on residents’ energy intake and the analysis of differences in energy intake of different age groups have important reference significance for the formulation of dietary nutrition security policies for residents in developing countries.

At present, the population structure of Chinese society is undergoing great changes. In 2022, the total population recorded negative growth for the first time in nearly 60 years. In the context of the continuous change in China’s population structure, the impact of demographic factors on the nutritional intake of Chinese residents cannot be ignored [9,10]. With the gradual emergence of the follow-up effect of the family planning policy and the full implementation of the two-child policy, China’s population structure will undergo key changes in the future [11,12]. The new demographic structure features of “fewer children” and “aging” will have a significant impact on China’s food demand, which is directly related to the scale and type of China’s food imports [13,14]. Therefore, the demographic changes in China will have a long-term and profound impact on the food and nutritional needs of the people of China and the world [15,16].

**Figure 1:**
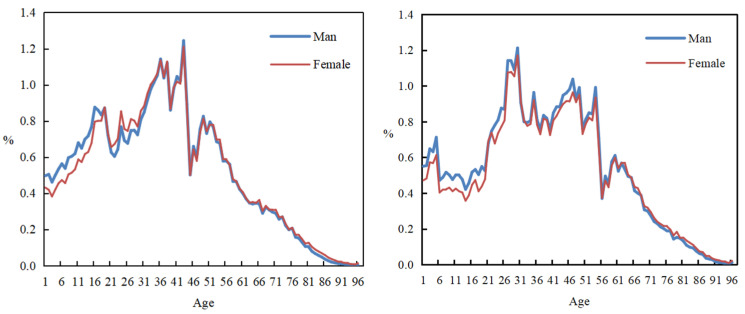
Comparison of the gender and age structure of China’s urban population in 2005 (**left**) and 2016 (**right**). Note: The vertical axis shows the percentage of the country’s population of different ages in the total population, expressed in %. Data from China Demographic Yearbook.

With a large number of rural residents moving to cities, the population structure of urban residents in China has undergone significant changes [17]. Figure 1 shows that the change in China’s urban population structure presents the characteristics of “fewer children” and “aging”. On the one hand, the proportion of the urban population aged 0–14 dropped from 20.27% in 2005 to 14.68% in 2016, indicating that China’s urban population has become a “fewer children” society. On the other hand, in 2005, the proportion of the urban population over the age of 65 exceeded the standard of 7%, which defines an aging society (according to the classification criteria set by the United Nations “Population Aging and Its Socio-economic Consequences” in 1956, when the number of elderly people aged 65 and above in a country or region accounts for more than 7% of the total population, it means that the country or region has entered the aging process), and continued to rise to 9.6% in 2016, indicating that China’s urban aging phenomenon has further deepened. At the same time, Chinese residents are becoming increasingly overweight and obese. According to the report on Nutrition and Chronic Diseases in China 2020 [18], the overweight and obesity rate of Chinese adult residents exceeds 50%, while it is nearly 20% in children and adolescents aged 6–17, and 10% in children under 6 years old. Studies have shown that dietary obesity caused by the imbalance between energy intake and physical activity is an important reason for the increasing incidence of obesity and chronic diseases in China [19,20]. In this context, the relationship between the change in China’s population structure and the change in residents’ energy intake has attracted more and more attention from the government and academia.

Approaches to studying the impact of demographic changes on consumption can be summarized into two categories. One method is to directly introduce the number or proportion of the population in different age ranges into the consumption function model as explanatory variables [21,22]. Tina and Yu [23] and You et al. [24] measured the impact of demographic changes on nutrition intake in Chinese families by adding the proportion of people in different gender and age groups into the nutrition demand equation. Xin et al. [25] analyzed the relationship between the age change in the Chinese population and the consumption of rations by using the data from the China Health and Nutrition Survey (CHNS). The study showed that with the increase in individuals’ age, meat consumption showed a trend of first increasing, then stabilizing, and then decreasing, while eggs did not show significant differential consumption characteristics.

Another method is to construct a comprehensive index to characterize the population structure and then introduce it into the consumption function. Among them, the adult equivalent scale method is a method for constructing the representation index of population structure [26,27,28]. Wang and Fu [29] used the adult equivalent scale method to construct a comprehensive variable of population structure by converting the food consumption of children and the elderly into the proportion of adult food consumption and studied the impact of an aging population on consumption. Gould [30] used the concept of the adult equivalent scale to construct an equal adult consumption weight model, construct a comprehensive index representing the population structure, and estimate the impact of population structure changes on the domestic food consumption expenditure of urban residents in China.

Using the adult equivalent scale method, the equivalent adult consumption weight of different age groups can be successfully converted, and then, the change in individual consumption in the whole life cycle can be observed. There are two ways to obtain adult equivalent scale values for different populations. One is to obtain adult equivalent scale values based on nutrient requirements [31]. For example, Zhong et al. [28] obtained equivalent adult consumption weights for different populations according to the table of individual daily caloric demand provided by Food and Agriculture Organization of the United Nations (FAO). The other is to use adult equivalent scale models and consumption data to estimate equivalent adult consumption weights [30,32]. Li and Chen [33] used the data on food consumption expenditure of Chinese urban residents and the adult equivalent scale model to reveal the changes in the adult equivalent scale of food consumption expenditure of different types of households. The results show that compared with non-only-child families, the adult equivalent scale of the food expenditure of members of only-child families is relatively higher, and this difference is particularly obvious for children under 17 years of age. In addition, some people set the equal adult consumption weight for different populations subjectively. Although the consumption power of people in different age groups can be distinguished, the human subjective factor is added [25].

Adult equivalent scale estimation using scale models and consumption data has a wide range of applications. First, the standard consumer size at the household level or national level can be calculated by using the adult equivalent of consumption of different populations, which is often applied to population size forecasting and food demand forecasting studies [8]. Second, it can depict the change in individual consumption levels throughout the whole life cycle, which provides convenience for analyzing the change in consumption. In addition, this approach reduces the bias that comes with subjectively setting adult equivalents.

In view of the above analysis, this study focuses on two research questions. First, to what extent the change in Chinese family demographic structures has an impact on family energy intake. The second is how the energy intake of residents changes with age in the whole life cycle. Just as the developed countries of East Asia, Japan and South Korea, experienced declining birth rates and a large proportion of elderly people, China’s society will inevitably undergo similar demographic changes as it rapidly moves toward the ranks of advanced countries. In recent years, China’s population has experienced negative growth for the first time, and the number of elderly people has increased rapidly. This demographic change will not only have a significant impact on China’s food consumption and nutritional intake, but also have a certain impact on world food demand and international trade. Therefore, the main contribution of our research is to understand how people’s energy intake changes with age in developing country economies with a large population base, demographic policy transformation, and rapid income growth, which can provide a reference for the formulation of nutrition security policies for people in other developing countries around the world. At the same time, in terms of academic contributions, we use the equivalent scale method to analyze the impact of family structure changes on residents’ energy intake, which can provide a reference for the analysis of the impact of population structure changes. We believe that if the effects of changes in household demographic structure are incorporated into the study of food demand and nutritional consumption by using the adult equivalent scale method, the accuracy of food demand prediction, the validity of poverty line measurements, and the accuracy of inter-household welfare comparisons can be greatly improved.

## 2. Materials and Methods

### 2.1. Analysis Framework

**Figure 2:**
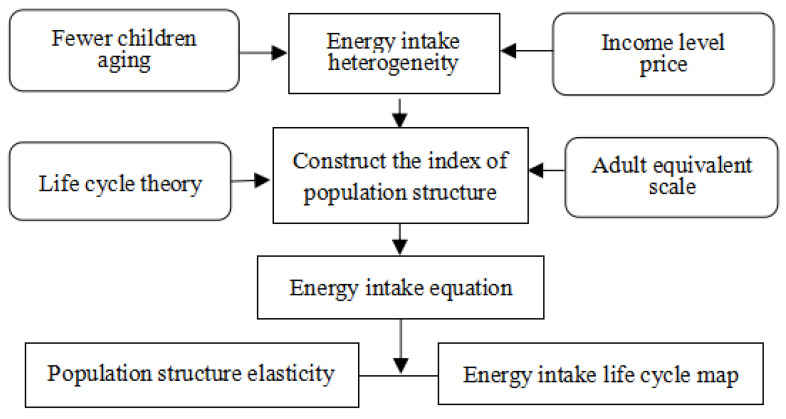
Analysis framework.

Figure 2 provides research paths and analysis ideas. Based on the life cycle theory, this paper constructs an adult equivalent scale model by using the concept of adult equivalent scale and the cubic spline function principle and constructs the representational variables of gender and age structure of family population. Then, the population structure representation variable is introduced into the energy intake equation as an independent variable. On the one hand, this paper empirically studies the impact of population structure change on energy intake. On the other hand, energy intake data are used to calculate the adult equivalent scales of different populations.

In this study, the least square estimation method and the instrumental variable estimation method were used to estimate the energy intake equation, respectively, and the stability of the estimated parameters was tested by comparison. Through a series of hypotheses, the significance of differences in energy intake levels between sex–age populations was examined. By calculating the elasticity of energy intake, the influence degree of population age structure and other factors on energy intake was estimated. The adult equivalent scales of energy intake in each age range were calculated by using the estimated parameter. The changes in energy intake in the life cycle with age were described using the graph. The difference between the energy scale and the population size was further compared in order to provide a reference for the calculation standard of population size.

### 2.2. Model Setting

#### 2.2.1. Construction of Variables Representing Population Structure

The adult equivalent scale is an alternative method for studying the effects of household demographics on food and nutrition consumption. The adult equivalent scale can standardize a population with different characteristics, so as to identify the differences in the impact of individuals with different characteristics on household consumption. That is, the weight of a standard person is set to 1, and then different weights are assigned to other people according to their gender and age characteristics, which can be used as scale values corresponding to different populations. Compared with some previous methods to study the impact of family demographics on consumption, the advantage of the adult equivalent scale is that it can capture the size effect of a large family and the difference in the cost of living between children and adults, and then, it can examine the impact of each family member on household consumption.

The development of adult equivalent scale has undergone a change from discrete to continuous. At the earliest, the adult equivalent scale function is set as a continuous function of the age and gender of family members, which solves the problem of discrete scale discontinuity. Then, based on psychological research and the concept of life cycle development, the age stratification of life cycle in the scale model is refined. The continuous adult equivalent scale has a more accurate definition of age stratification and is relatively reasonable, which is also related to the combination of psychological knowledge, child development, adult development, and other concepts in the establishment of the equivalent scale. Later, continuous adult equivalence scales were widely used to measure equivalence scales [33,34,35]. The use of adult equivalent scaling method has some limitations: one is that it requires a large amount of data to be used, and the other is to solve the econometric problem of introducing an adult equivalent scaling model into a regression model to estimate many unknown parameters. However, there are relatively few studies on Chinese dietary nutrition using the adult equivalent scale method. If we can take the energy intake of Chinese residents as the research object and use the adult equivalent scale method to measure the adult equivalent scale of individual energy intake and reveal the change trend of energy intake of Chinese residents throughout their life, it is of great significance to understand the change in energy intake of Chinese residents during the population transition period. The family data sample used in this study is large enough and the life cycle in the scale model is divided, which means that we can better calculate the adult equivalent scale corresponding to different age groups.

We construct equal standard consumption weights based on a person’s age and gender. We first set a 35–40-year-old male as the reference standard consumer, whose standard consumption weight is 1; his age is expressed as *a*_r_, and his gender is expressed as *g_r_*. Let *S_ij_* represent the equal standard consumption weight of the j family member in the *i* family, expressed as

*S_ij_* = *S* (*a_ij_*, *g_ij_*|*a_r_*, *g_r_*) (1)

*a_ij_* and *g_ij_* indicate the age and gender of the family members, respectively. The meaning of *S_ij_* is the equivalent standard consumption weight of an individual whose age is *a_ij_* and sex is *g_i_* calculated under the consumption conditions of the reference standard consumer.

Dividing the life cycle into nine stages based on life expectancy in China, these are newborn infants (*a_ij_* = 0), child or adolescent development stage (0 < *a_ij_* ≤ 17), youth transition stage (17 < *a_ij_* ≤ 22), youth development stage (22 < *a_ij_* ≤ 35), middle-age transition stage (35 < *a_ij_* ≤ 40), middle-age development stage (40 < *a_ij_* ≤ 55), middle- and old-age transition stage (55 < *a_ij_* ≤ 60), middle- and old-age development stage (60 < *a_ij_* ≤ 70), and old-age transition stage (*a_ij_* > 70), as shown in Table 1.

We use the construction principle of cubic spline function to calculate the concrete form of *S*(*a_ij_*, *g_ij_*) at each age. We take the formula for calculating the equal standard consumption weight of male members in the age group 0 < *a_ij_* ≤ 17 as an example, and the calculation process is as follows:

**Hypothesis** **1.** *S (0, M) = S (0, F) = c_1_. This means that the equal standard consumption weight of a newborn baby is set to c1, where M is male and F is female.*

**Hypothesis** **2.** *Both the first and second derivatives of the function S(a_ij_, g_ij_) with respect to a_ij_ exist, and the upper first and second derivatives are 0 in the transition stage, that is, when 17 < a_ij_ ≤ 22, 35 < a_ij_ ≤ 40, 55 < a_ij_ ≤ 60, a_ij_ > 70, it is satisfied.*

**Hypothesis** **3.** *S (17, M) = c_2_, S (35, M) = c_3_, S (55, M) = c_4_, S (70, M) = c_5_, S (17, F) = c_6_, S (35, F) = c_7_, S (55, F) = c_8_, S (70, F) = c_9_.*

According to the above assumptions, the formula *S*(*a_ij_*, *M*) of equal standard consumption weight for male members at the age of 0 < *a_ij_* ≤ 17 is assumed to be a cubic polynomial of age, expressed as

(2) $$S\left(a_{ij},M\right)=e_{01}+e_{11}a_{ij}+e_{21}a_{ij}^{2}+e_{31}a_{ij}^{3}$$

where *a_ij_* represents the age of the *j* family member in the *i* family, *M* indicates that the member is male, and *e*_01_, *e*_11_, *e*_21_, and *e*_31_ are unknown coefficients.

According to hypothesis 1, the equal adult consumption weight of a newborn baby is *c*_1_; then, it can be obtained from the continuity of the cubic spline function that

(3) $$S\left(0,M\right)=e_{01}=c_{1}.$$

According to Hypothesis 3, *S* (17, *M*) = *c*_2_; then, the continuity of the cubic spline function can be obtained:

(4) $$S\left(17,M\right)=e_{01}+17e_{11}+17^{2}e_{21}+17^{3}e_{31}=c_{2}$$

From Hypothesis 2 and the continuity of the function, we know that the first derivative of *S*(*a_ij_*, *M*) with respect to *a_ij_* is zero at age 17.

(5) $$\partial S(a_{ij},M)/\partial a_{ij}{|}_{a_{ij}=17}=e_{11}+34\times e_{21}+867\times e_{31}=0.$$

By Equations (3)–(5), *e*_21_ and *e*_31_ are represented by *c*_1_, *c*_2_ and *e*_11_, and then substituted into Equation (2), and the equivalent standard consumption weight formula of male members in the age range of 0 < *a_ij_* ≤ 17 is obtained, as shown below.

(6) $$S\left(a_{ij},M\right)=c_{1}+e_{11}a_{ij}-\left[0.1176e_{11}+0.0104\left(c_{1}-c_{2}\right)\right]a_{ij}^{2}+[0.0035e_{11}+0.0004\left(c_{1}-c_{2}\right)]a_{ij}^{3}.$$

where *a_ij_* is the age, *c*_1_ is the equal standard consumption weight of newborn infants, *c*_2_ is the equal standard consumption weight of 17 < *a_ij_* ≤ 22 males, and *e*_11_ is the unknown parameter. Using this calculation process, the standard consumption weight formulas of child or adolescent development stage (0 < *a_ij_* ≤ 17), youth development stage (22 < *a_ij_* ≤ 35), middle-age development stage (40 < *a_ij_* ≤ 55), and middle- and old-age development stage (60 < *a_ij_* ≤ 70) are calculated, respectively.

Table 1 reports the specific forms of equal standard consumption weights for different ages or age groups. Among them, *c*_3_ = 1. *c*_1_, *c*_2_, *c*_4_, *c*_5_, *c*_6_, *c*_7_, *c*_8_, *c*_9_, *e*_11_, *e*_21_, *e*_31_, *e*_41_, *e*_12_, *e*_22_, *e*_32_, and *e*_42_ are unknown parameters.

The equal adult consumption weights corresponding to each age interval in Table 1 are added together to obtain the adult equivalent scale of energy intake (*EAES*), a characteristic variable representing population structure, expressed as

*EAES* = *S*_0_ × *n*_0_ + *S*_1_ × *n*_1_+…+*S*_16_ × *n*_16_ (7)

*S_0_*, *S*_1_,…, *S*_16_ is equal adult consumption weight of the population of each age group. *n*_0_, *n*_1_,…, *n*_16_ is the number of population by age group. We put the specific form of the equal adult consumption weight formula *S*(*a_ij_*, *g_ij_*) in Table 1 into Equation (7) to obtain the *EAES* variable composed of unknown parameters *c*_1_ − *c*_9_, *e*_11_, *e*_21_, *e*_31_, *e*_41_, *e*_12_, *e*_22_, *e*_32_, and *e*_42_. We put the *EAES* variable, which represents the household population structure, into the energy consumption function model, and estimate the unknown parameters using the micro-household energy consumption data. When the unknown parameters in Table 1 are estimated, adult equivalent consumption weights, such as energy consumption for each age group, are determined.

#### 2.2.2. Construction of Variables Representing Population Structure

According to the definition of health production function by Thomas [36], nutrient intake is a function of a series of input factors that meet the maximum utility under the constraints of household budget. Therefore, the specific model form of the energy intake equation is set as follows:

(8) $$N=\alpha_{0}+\alpha_{1}I+\alpha_{2}P+\alpha_{3}D+\alpha_{4}EAES+\mu$$

where *N* is the daily household energy intake; *I* is family income; *P* is the food price index; and *EAES* is a variable representing the family demographic structure. *D* is the household characteristic variable that affects nutrition intake, including the education level of the head of household, the proportion of food expenditure outside the home, city size, household status, and regional variables. Regional dummy variables included Hebei, Henan, Sichuan, Guangdong, and Xinjiang, with Xinjiang as the reference group. $\alpha_{0},\alpha_{1},\alpha_{2},\alpha_{3},\alpha_{4}$ is an unknown item; μ is a random error. The *EAES* variable contains the unknown parameters *c*_1_ − *c*_9_, *e*_11_, *e*_21_, *e*_31_, *e*_41_, *e*_12_, *e*_22_, *e*_32_.

Considering that the bidirectional relationship between energy intake and income may lead to endogeneity problems in an energy intake model, instrumental variable estimation method is used to solve the endogeneity problem of income. By summarizing previous research, the possible instrumental variables to solve the endogeneity of income can be summarized as the characteristics of the working population in the household, the years of education of the household head, non-food expenditure (expenditure on clothing, housing, durable consumer goods, medical care and health, culture, education and entertainment, etc.), household housing conditions, etc. [23,37]. In view of previous studies and the availability of the data used, the share of the working population in the household, the amount of durable goods (household equipment, vehicles, recreational goods) spent, and the amount of other real estate were selected as possible combinations of instrumental variables for income. In general, the larger the number of working people in a household, the larger the number of houses in a household, and the higher the expenditure on durable goods, the higher the household income level tends to be, and these variables are not particularly strongly correlated with the energy intake level of residents.

In order to estimate the degree of influence of influencing factors on energy intake, energy intake elasticity E was calculated, which is expressed as follows:

(9) $$E=(\partial N/\partial X)(\bar{X}/\bar{N})$$

Here, *X* represents the influencing factors of energy intake. ∂N/∂X is the first derivative of energy intake to the influencing factors in the energy intake equation. $\bar{X}$ is the average value of the influencing factors. $\bar{N}$ is the average value of energy intake. The value of elasticity *E* represents the percentage change in energy intake caused by a 1% change in the influencing factor.

### 2.3. Data Description

The data used are from the urban household survey data of the National Bureau of Statistics of China in 2009. The National Bureau of Statistics of China has not published urban household survey data since 2009, so the data of this paper are representative, and many previous studies have used such survey data [13,38]. The urban Household Survey team of the National Bureau of Statistics of China obtained the urban household survey data through sampling surveys. According to the survey plan, urban households refer to households within the administrative areas of the urban areas and the resident committees of counties and towns, including permanent non-agricultural households registered in the local area, permanent agricultural households registered in the local area, non-agricultural households registered in other places and residing in the local area for more than six months, and agricultural households registered in other places and residing in the local area for more than six months. The sample households in the survey kept a daily record of their income and expenses for an entire year. The samples used in this paper are the remaining samples after the outliers are removed from the randomly selected urban household data. We selected 10,462 urban households representing the six provinces of North, South, Central, Southwest, and Northwest China: Hebei (1935 households), Guangdong (2371 households), Henan (1602 households), Jilin (1222 households), Sichuan (2246 households), and Xinjiang (1086 households).

The data included household food consumption at home and spending on food away from home. The process of obtaining energy intake data is as follows: First, determine the m main foods consumed in the family, and set ten main food groups here. They are grains (rice, flour), fats (animal fats, vegetable oil), meat (pork, beef, lamb), poultry (chicken, duck), eggs (eggs, duck eggs), aquatic products (fish, shrimp), dairy products (fresh dairy products, milk powder, yogurt), vegetables (fresh vegetables), fruits (fresh fruits, fresh melons), potatoes (starch, potatoes). Set *NI* as the energy intake from food consumption in the household, *NI* = $\sum_{k=1}^{m}$ *F_k_* × *Q_k_*, where *F_k_* represents the energy content contained in the k food, *k* = 1,…, *m*; *Q_k_* represents the consumption of food number *k*. Energy content data were obtained from the Nutrition Facts Table of Chinese Food provided by Nutrition and Food Safety, Chinese Center for Disease Control and Prevention [39]. Secondly, the proportion of energy intake from food consumption within the household to the total expenditure of *k* foods *R_k_* was calculated. This ratio was used to convert *NO* energy intake from food consumption outside the home and other food consumption [40]. Finally, *NI* and *NO* were added together to obtain the total household energy intake *N*.

### 2.4. Statistical Description of Variables

Household daily energy intake was used as the explained variable. As can be seen from Table 2, the average daily energy intake of the sample households was 6949 calories. Income level is an important factor affecting nutrition demand, and the average disposable income of a sample family is CNY 44,435.27. The education level is closely related to the kind of food that people choose to consume, which in turn affects the energy demand, so the independent variable includes the education level of the household head. Here, 41.8% of the sample households had a head of household with an education level of junior college or above. Residents with local household registration enjoy preferential treatment in terms of job opportunities and social security, which may have an impact on family food consumption, so the independent variable is added to the dummy variable of whether the household head is a local household registration. A total of 96.6% of the households in the sample were local households. The level of urbanization affects the convenience of households when buying food, so the control variable adds the dummy variable of whether households live in small cities. We set the county administrative areas as small cities. In total, 18.3% of the families in the sample live in small cities.

Urban residents eat out frequently, which may have an important impact on residents’ energy intake. We used the proportion of household food expenditure to reflect the impact of eating out on household energy intake. From the statistical description, the average proportion of the sample families’ dining out expenditure is 16%. Food prices affect the quantity and quality of food purchased. We chose a sample of households whose Stone Price Index variable reflects the price of food. The specific calculation process is as follows: Firstly, the price of each food group is calculated by using the expenditure and quantity of ten main food groups consumed by each family; then, the corresponding food price index variable for each household is calculated according to the Stone Price Index formula [27]. The average stone price index for the sample of households was CNY 9.476 per kg. According to the description of the regional variables of the samples, the number of families from Henan, Hebei, Jilin, Guangdong, Sichuan, and Xinjiang accounted for 15.3%, 18.5%, 11.7%, 22.7%, 21.5%, and 10.4% of the total samples, respectively. The proportion of the sample number in each province exceeded 10%, indicating that the sample selection was highly representative. According to the statistics of instrumental variables, the average proportion of working population in the sample households was 60.8%, the average expenditure on household durable goods (household equipment, transportation, cultural, and entertainment products) was CNY 9730.95, and the average number of other houses was 0.118 sets.

## 3. Results

### 3.1. Classification and Comparative Analysis of Resident Energy Intake

**Figure 3:**
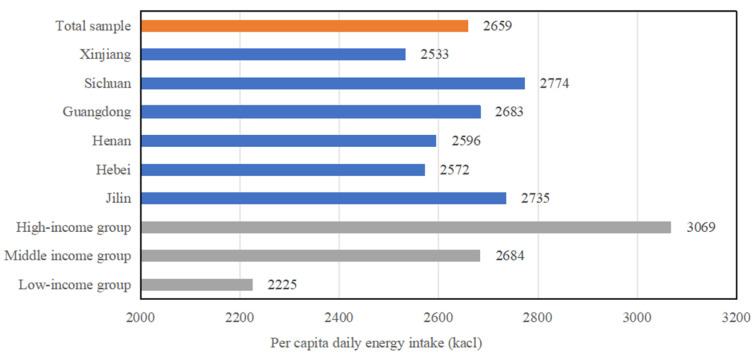
Per capita energy intake of urban residents in different provinces and income groups. Note: 1. The target range of energy intake given in the Outline of Food and Nutrition Development in China (2014–2020) is 2200–2300 calories. 2. The recommended intake range from light to high activity level for men and women aged 18–50 provided in the Dietary Nutrient Reference Intake for Chinese Residents (2013 edition) is 2250–3000 calories for men aged 18–50; 1800–2100 calories for women aged 18–50.

As can be seen from Figure 3, the per capita daily energy intake of the total sample was 2659 calories, and the per capita daily energy intakes of urban residents in Jilin Province, Guangdong Province, and Sichuan Province were higher than the intake level of the total sample, while those of Hebei Province, Henan Province, and Xinjiang Province were lower than the intake level of the total sample. Compared with the nutritional standards, the energy intake of the total sample and the samples of provinces and cities were higher than the nutritional target intake (2200–2300 calories) given in the Outline of Food and Nutrition Development of China (2014–2020) [41]. However, all of them were within the recommended energy intake range for men (2250–3000 calories) set by the Dietary Nutrient Reference Intake for Chinese Residents (2013) [42].

The per capita daily energy intake rises with the increase in income group. The average daily energy intake of the high-income group was 3069 kcal, which was 385 kcal higher than that of the middle-income group and 843 kcal higher than that of the low-income group. Compared with the standards of the Outline of Food and Nutrition Development of China (2014–2020), the energy intake of the low-income group was within the nutritional target, and the energy intakes of the middle-income group and the high-income group were greater than the nutritional target.

### 3.2. Population Structure Factors That Significantly Affect the Energy Intake of Urban Residents

Table 3 shows the least squares (OLS) and instrumental variable (IV) estimates of the energy intake. According to the results of the endogeneity test of DWH estimated by IV, the significance level of the F value is 1%, indicating the existence of income endogeneity in the energy intake equation. In order to solve the endogeneity of income, the characteristics of the working population in the household, the expenditure of household durable goods, and the amount of real estate are selected as possible instrumental variables. Different combinations of instrumental variables were tried, and the F-test and Sargant test were used to test the validity of the instrumental variables. Finally, the proportion of working population in the family and the number of other houses in the family were selected as the instrumental variables of income in the energy intake equation. From the validity test results of instrumental variables, the F statistic for testing weak instrumental variables is greater than 10, and the Sargan statistic for testing the endogeneity of instrumental variables is not significant, indicating that the selected instrumental variables are valid (Table 3). To avoid possible heteroscedasticity problems, all models estimate the robustness standard error. The following takes the estimated results of the IV model as the main analysis object.

Most of the parameters in the results of the IV model are estimated significantly, which indicates that the model estimates well. Except for the education level of the head of household and Jilin Province variables, the statistical significance level of the other economic and social variables is below 5%. Among the 17 parameters to be estimated in the EAES variable, 15 have an estimated significance level below 5%, which also indicates that the age structure of the family has a significant impact on the family energy intake.

According to the results of the parameters to be estimated that are contained in the EAES variable in the IV model, a series of Wald tests were conducted on the parameters of the age structure variable, and the results are provided in Table 4. First, all age structure variable parameters were significantly combined, and the age structure variable parameters of male and female were significantly combined, indicating that the population age structure had a significant impact on the family’s energy intake. Second, there were differences in the adult equivalent scale of energy intake between men and women in the four age groups. According to the adult equivalent scale formula in Table 1, the estimated values of parameters *c*_2_, *c*_3_, *c*_4_, and *c*_5_ correspond to the adult equivalent scales of energy intake for males aged 17–22, 35–40, 55–56, and over 70, respectively. Estimates of parameters *c*_6_, *c*_7_, *c*_8_, and *c*_9_ correspond to the adult equivalent scales of energy intake for women aged 17–22, 35–40, 55–56, and over 70 years, respectively. On this basis, a hypothesis test was conducted to test whether there are differences in adult equivalence scales between males and females in different age groups. From the test results, there were differences in the adult equivalent scales of energy intake between men and women in the four age groups.

In the results of the IV estimation, the estimated coefficients of the impact of household disposable income on energy intake are significantly positive, and the estimated coefficients of food price are significantly negative, indicating that there is a positive relationship between the household income and energy intake and a negative relationship between food prices and energy intake. An important finding is that the variable of the proportion of spending on eating out is estimated to be significantly negative. This indicates that although urban families eat out frequently and spend more money, it does not necessarily lead to an improvement in energy intake. Residents generally spend more on eating out, but often, the high price is related to the appearance and taste of the food consumed, which leads to the fact that the energy contained in the food consumed out may not be as high as that contained in the food consumed at home. The variable of whether the household is in a small city is estimated to be significantly negative, indicating that the energy intake of small-city households is relatively smaller than that of large city households. According to the results of the regional variable estimation, the parameter estimates of variables in Henan and Hebei Province are significantly negative, while those in Guangdong and Sichuan Province are significantly positive, indicating that there are significant differences in household energy intake between these provinces and the reference group in Xinjiang.

### 3.3. Household Energy Intake Is More Responsive to Changes in Population Structure

**Figure 4:**
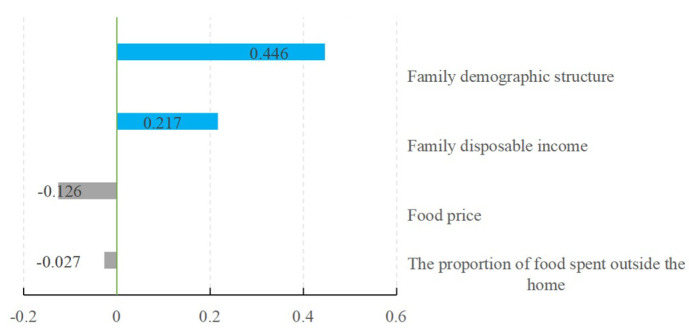
Elastic results of energy intake.

According to the parameter estimation results of the IV model, the elasticity of continuous variables is calculated, and the results are summarized in Figure 4. The important finding is that the EAES variable has an elasticity value of 0.446 for household energy intake with a significance level of 1%. The results show that a 1% change in the household population structure variable, represented by equivalent adult consumption weight, leads to a 0.446% change in household energy intake.

Notably, the income elasticity of household energy intake was 0.217 with a significance level of 1%. The results show that household income significantly affects household energy intake. When the household income increases by 1%, the household energy intake increases by 0.217%. The elasticity of the proportion of food expenditure on household energy intake is negative, with a value of −0.027 and a significance level of 1%. This shows that there is a negative relationship between eating out and household energy demand. One possible explanation is that people may pay more attention to nutrition when eating at home, while when they are eating out, they may pay more attention to taste, and food that tastes good does not necessarily have a high nutritional value. Food prices negatively affect energy demand, which is in line with theoretical expectations. The food price elasticity of household energy intake is −0.126, and the significance level is 1%, indicating that when the food price increases by 1%, the household energy intake decreases by 0.126%.

### 3.4. Residents’ Energy Intake Varies Significantly over the Life Cycle

**Figure 5:**
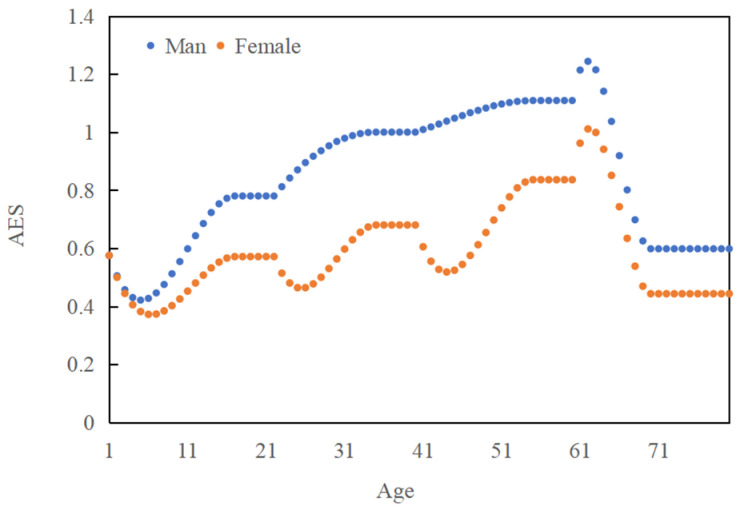
The variation in the adult equivalent scale (AES) of energy intake over the whole life cycle.

The life cycle changes in energy intake in Chinese residents show the following characteristics: First, there are differences in the adult equivalent scale of energy intake between males and females. As can be seen from Figure 5, the adult equivalent scale of energy intake for males is always greater than that for females.

The important finding is that with the increase in age, the energy intake of elderly people over 60 years of age presents a trend of first an increase and then a decrease, and especially after the age of 65 years, the decline trend is very obvious. The reason is that with the increase in age, the health awareness of the elderly over 60 years increases, and they care more about their health, resulting in a preference for buying higher quality and safer food and increasing their energy intake. However, with the further increase in age, the energy intake of people over the age of 65 rapidly drops to very low levels. This also suggests that as the proportion of elderly people over the age of 65 continues to increase in China’s urban population, it will drive down China’s food consumption.

It is worth noting that the population in the 55–64-year age range has a relatively high energy intake compared to other age groups. Two reasons can account for this phenomenon. First, the retirement age for Chinese women and men was 50 and 55, respectively, in 2009. When they retire, they become more health-conscious and increase their purchases of foods with high nutritional content. The second is that retired people have more leisure activities, including more calorie-consuming activities such as square dancing, which may increase their energy consumption. Therefore, age, retirement policy, health awareness, and lifestyle should be taken into account when analyzing the changes in energy demand of retired people.

The interesting finding is that the energy intake of children in the age range of 1–10 years showed first a decline and then an increase. One possible explanation is that, as a result of China’s early implementation of the family planning policy, infants and young children receive extra attention from their parents and are supplied with more energy-rich nutrients, such as high-energy formula. As children get older, parental care decreases, and children’s energy intake briefly declines. After about 4 years of age, with the needs of growth and development, children’s energy intake will gradually increase. In addition, the energy intake of women in the age range of 22–30 years and 40–55 years also showed first a downwards and then an upwards change. Generally speaking, after the age of 21, Chinese women enter the legal marriage age, and they may pay attention to their diet to maintain a better figure, which may lead to a decline in energy intake. When the women in the family reach the age of 39, their children will have grown up and started to leave the family, which gives the women more time to work on body management, and there is a decrease in energy intake to maintain health.

**Figure 6:**
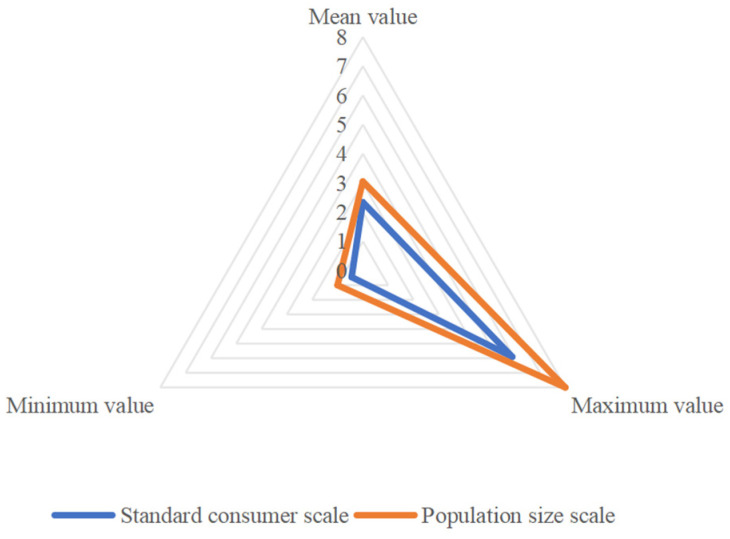
Family size expressed by different criteria.

There are differences between the two kinds of family size expressed by the adult equivalent scale of energy intake and the population size, respectively. The average household size measured by energy intake was 2.341 standard consumers, while the average household size expressed by population was 3.052 people (Figure 6). The reason for the difference is that the family size expressed by the artificial scale of the standard consumption of energy intake includes the gender and age structure of the family members. However, the assumption behind the total number of family members is that each family member is undifferentiated.

## 4. Discussion

The important finding is that the energy intake of Chinese elderly over 60 years old shows a trend of first increasing and then decreasing, especially after the age of 65 years. This indicates that with the continuous increase in the proportion of the population over the age of 65 in Chinese cities and towns, it may lead to a great change in the consumption structure of food and nutrition in China, that is, aging promotes a decline in food consumption in Chinese society. As for the impact of aging on the food consumption of Chinese residents, most studies believe that there will be a rapid decline in food consumption after the age of 60, which is called the “Retirement Consumption Puzzle” [43,44]. The reason for the precipitous decline in food consumption is that retirement at the age of 60 leads to a drop in income, which in turn leads to a decline in food consumption. Our results from the energy perspective show that the energy intake level of Chinese elderly over the age of 60 will not decline rapidly but will first have an upward trend. Our analysis shows that with the increase in age, elderly people over 60 years of age increase their health awareness, care more about their health, prefer to buy higher quality and safer food, and increase their energy intake. However, with the further increase in age, the energy intake of people over the age of 65 rapidly declines to very low levels.

The interesting finding is that the energy intake of children in the age range of 1–10 years, women in the age range of 21–30 years, and women in the age range of 39–49 years all showed a decrease and then an increase. Taking into account the current situation of family development in China, we conclude that with the increase in age, parents’ care for infants and toddlers gradually decreases, leading to a temporary decline in the energy intake of children aged 1–10 years. Changes in energy intake for women in the 21–30- and 39–49-year age ranges are attributed to body management, which reduces energy intake. These results are consistent with the current situation of family development in China. The family planning policy has led to the emergence of a large number of one-child families in China. As a result, one-child families pay more attention to the nutritional needs of infants and children and tend to buy foods that are rich in energy. As a result, infants have a higher energy intake. As children get older, parental care decreases, and children’s energy intake briefly declines.

The average household size expressed by the adult equivalent scale of energy intake was 2.341 standard persons, and the average household size expressed by the population was 3.052 persons. Compared with the size of the household population, the household size measured by the standard energy consumer contains more information about the sex and age of the household members and has important applications [27]. In view of this advantage, the adult equivalent scale can improve the accuracy of family size measurement and can be more widely applied to the comparison of inter-family welfare and the formulation of the poverty line [45].

The impact of demographic changes on household energy intake cannot be ignored. A series of hypothesis testing results show that all age structure variable parameters are significantly combined, the age structure variable parameters of male and female are significantly combined, and there are differences in adult consumption weights such as the energy intake of males and females at different ages, indicating that the population age structure has a significant impact on family energy intake. The elasticity of the *EAES* variable representing family population structure on energy intake is 0.446, indicating that the change in population structure representation variable is 1%, and the change in family energy intake is 0.446%. This conclusion implies that with the acceleration of “fewer children and aging”, the differences in energy consumption between men and women, food and nutrition demand forecasts, and simulations should be adjusted accordingly to account for these changes and differences, which will help in the development of food security strategies.

## 5. Conclusions

The adult equivalence scale established in this study only considers the age change of family members during their life cycle and ignores the influence of family type on family consumption behavior. Different types of families not only have different gender and age compositions of family members but also have different family decision making processes. These differences in family structure characteristics lead to great differences in dietary nutrient intake among families. In the late 1970s, China’s one-child policy led to the emergence of a family structure that is different from other countries, and one-child families have become the main body of urban families in China. Although the recent two adjustments of the two-child policy have pushed China into the “post-one-child era”, the birth policy has an obvious “lag”, and the possible impact of the comprehensive two-child policy will not appear until the two children gradually grow up. Therefore, the effect of the unique change in family structure brought by the one-child policy will exist for a long time. Compared with multi-child nuclear families, one-child families may have a certain uniqueness in their life cycle and consumption behavior because of the limit on the number of children that they are allowed to have. Our findings also show that Chinese families pay special attention to their children’s dietary nutrition. Therefore, in future studies, it is necessary to take into account the particularity of the development of Chinese family structure, pay attention to the differences in dietary nutrition of different types of families and the structural characteristics of one-child families, and establish an adult equivalent scale model that integrates the comprehensive systematic characteristics of individual life cycle and family life cycle.

In this paper, the adult equivalent scale model introduced the population structure representation variable into the consumption function, quantified the impact of household population structure changes on energy intake, and provided a more accurate method for analyzing the impact of family structure, which has enriched empirical research on the impact of population structure changes on consumption to a certain extent. At the same time, the household adult equivalent scale based on energy intake has important practical significance. Family size, expressed on the adult equivalent scale, takes into account differences in the gender and age characteristics of family members, while the assumption behind the total number of family members is that each family member is undifferentiated. Therefore, the energy intake level in a per capita sense can reflect the difference in family population structure by using the equivalent scale of adults in a family. This per capita energy intake can be applied to the comparison of dietary nutrition levels among families in developing countries, as well as to the comparison of welfare among families and the formulation of poverty lines.

## Figures and Tables

**Table 1:** Formula of equal standard consumption weights for different age and sex populations.

Weight Variable	Age	Equal Standard Consumption Weight Formula *S*(*a_ij_*, *g_ij_*)	Population Quantity
Newborn baby			
*S*_0_	*a_ij_* = 0	*c*_1_	*n*_0_
Male			
*S*_1_	0 < *a_ij_* ≤ 17	*c*_1_ + *e*_11_*a_ij_* − [0.1176*e*_11_ + 0.0104(*c*_1_ − *c*_2_)] *a_ij_*^2^ + [0.0035*e*_11_ + 0.0004(*c*_1_ − *c*_2_)] *a_ij_*^3^	*n*_1_
*S*_2_	17 < *a_ij_* ≤ 22	*c*_2_	*n*_2_
*S*_3_	22 < *a_ij_* ≤ 35	*c*_2_ + *e*_21_(*a_ij_* − 22) − [0.1538*e*_21_ + 0.0176(*c*_2_ − *c*_3_)](*a_ij_* − 22)^2^ + [0.0059*e*_21_ + 0.0009(*c*_2_ − *c*_3_)](*a_ij_* − 22)^3^	*n*_3_
*S*_4_	35 < *a_ij_* ≤ 40	*c*_3_ = 1	*n*_4_
*S*_5_	40 < *a_ij_* ≤ 55	*c*_3_ + *e*_31_(*a_ij_* − 40) − [0.1333*e*_31_ + 0.0133(*c*_3_ − *c*_4_)](*a_ij_* − 40)^2^ + [0.0044*e*_31_ + 0.0006(*c*_3_ − *c*_4_)](*a_ij_* − 40)^3^	*n*_5_
*S*_6_	55 < *a_ij_* ≤ 60	*c*_4_	*n*_6_
*S*_7_	60 < *a_ij_* ≤ 70	*c*_4_ + *e*_41_(*a_ij_* − 60) − [0.2*e*_41_ + 0.03(*c*_4_ − *c*_5_)](*a_ij_* − 60)^2^ + [0.01*e*_41_ + 0.002(*c*_4_ − *c*_5_)](*a_ij_* − 60)^3^	*n*_7_
*S*_8_	*a_ij_* > 70	*c*_5_	*n*_8_
Female			
*S*_9_	0 < *a_ij_* ≤ 17	*c*_1_ + *e*_12_*a_ij_* − [0.1176*e*_12_ + 0.0104(*c*_1_ − *c*_6_)] *a_ij_*^2^ + [0.0035*e*_12_ + 0.0004(*c*_1_ − *c*_6_)] *a_ij_*^3^	*n*_9_
*S*_10_	17 < *a_ij_* ≤ 22	*c*_6_	*n*_10_
*S*_11_	22 < *a_ij_* ≤ 35	*c*_6_ + *e*_22_(*a_ij_* − 22) − [0.1538*e*_22_ + 0.0176(*c*_6_ − *c*_7_)](*a_ij_* − 22)^2^ + [0.0059*e*_22_ + 0.0009(*c*_6_ − *c*_7_)](*a_ij_* − 22)^3^	*n*_11_
*S*_12_	35 < *a_ij_* ≤ 40	*c*_7_	*n*_12_
*S*_13_	40 < *a_ij_* ≤ 55	*c*_7_ + *e*_32_(*a_ij_* − 40) − [0.1333*e*_32_ + 0.0133(*c*_7_ − *c*_8_)](*a_ij_* − 40)^2^ + [0.0044*e*_32_ + 0.0006(*c*_7_ − *c*_8_)]*a_ij_* − 40)^3^	*n*_13_
*S*_14_	55 < *a_ij_* ≤ 60	*c*_8_	*n*_14_
*S*_15_	60 < *a_ij_* ≤ 70	*c*_8_ *+ e*_42_(*a_ij_* − 60) − [0.2*e*_42_ + 0.03(*c*_8_ − *c*_9_)](*a_ij_* − 60)^2^ + [0.01*e*_42_ + 0.002(*c*_8_ − *c*_9_)](*a_ij_* − 60)^3^	*n*_15_
*S*_16_	*a_ij_* > 70	*c*_9_	*n*_16_

**Table 2:** Statistical description of variables.

Variable	Total Sample	Standard Deviation	Minimum	Maximum
Daily household energy intake (kcal)	6949	2757.857	719	22,587
Household disposable income (CNY)	44,435.27	26,063.39	1820	238,163
Education level of head of household (1 = college and upper level, 0 = other)	0.418	0.493	0	1
Account status (1 = local account, 0 = other)	0.966	0.182	0	1
City size (1 = small city, 0 = other)	0.183	0.386	0	1
Proportion of spending on eating out (%)	0.16	0.145	0	0.933
Food price (CNY/kg)	9.476	2.958	3.162	28.349
Henan (1 = yes, 0 = other)	0.153	0.360	0	1
Hebei (1 = yes, 0 = other)	0.185	0.388	0	1
Jilin (1 = yes, 0 = other)	0.117	0.321	0	1
Guangdong (1 = yes, 0 = other)	0.227	0.419	0	1
Sichuan (1 = yes, 0 = other)	0.215	0.411	0	1
Xinjiang (1 = yes, 0 = other, reference group)	0.104	0.305	0	1
Instrumental variable (as follows)				
Durable goods expenditure (CNY)	9730.95	14,119.14	0	274,162.6
Percentage of people working in households	0.608	0.308	0	1
Number of other properties (units)	0.118	0.366	0	6
Sample size	10,462			

**Table 3:** Estimated results of energy intake equation.

Variable and Parameters	OLS Model		IV Model	
	Coefficient	Standard Error	Coefficient	Standard Error
Logarithm of household disposable income	0.325 ***	0.008	0.217 ***	0.033
Head of household education level	−0.049 ***	0.008	−0.017	0.012
Household registration status	0.070 ***	0.019	0.054 ***	0.020
City size	−0.077 ***	0.009	−0.067 ***	0.010
Proportion of spending on eating out	−0.280 ***	0.026	−0.166 ***	0.043
Logarithm of food price	−0.172 ***	0.020	−0.126 ***	0.024
Henan	−0.108 ***	0.014	−0.082 ***	0.017
Hebei	−0.125 ***	0.014	−0.096 ***	0.017
JiLin	−0.023	0.015	−0.009	0.016
Guodong	0.019	0.017	0.043 **	0.018
Sichuan	0.071 ***	0.014	0.073 ***	0.014
α_4_ × *c*_1_	0.125 ***	0.025	0.128 ***	0.025
α_4_ × *c*_2_	0.146 ***	0.010	0.149 ***	0.010
α_4_ × *c*_3_ (*c*_3_ = 1)	0.155 ***	0.014	0.191 ***	0.018
α_4_ × *c*_4_	0.175 ***	0.016	0.212 ***	0.019
α_4_ × *c*_5_	0.081 ***	0.016	0.114 ***	0.019
α_4_ × *c*_6_	0.105 ***	0.010	0.109 ***	0.010
α_4_ × *c*_7_	0.113 ***	0.015	0.130 ***	0.016
α_4_ × *c*_8_	0.136 ***	0.016	0.159 ***	0.018
α_4_ × *c*_9_	0.067 ***	0.015	0.084 ***	0.016
α_4_ × *e*_11_	−0.020 **	0.009	−0.021 **	0.009
α_4_ × *e*_21_	0.001	0.008	0.006	0.008
α_4_ × *e*_31_	−0.001	0.006	0.002	0.006
α_4_ × *e*_41_	0.029 **	0.012	0.028 **	0.012
α_4_ × *e*_12_	−0.019 **	0.009	−0.021 **	0.009
α_4_ × *e*_22_	−0.019 **	0.007	−0.013 *	0.008
α_4_ × *e*_32_	−0.015 **	0.006	−0.017 ***	0.006
α_4_ × *e*_42_	0.033 **	0.013	0.032 **	0.013
Constant term	5.371 ***	0.081	6.310 ***	0.294
Endogeneity test: DWH test			11.319 ***	
Weak instrumental variable test: F test			287.778 ***	
Sargan test			1.533	
Observed value	10,462		10,462	
Goodness of fit	0.296		0.282	

Note: ***, **, and * denote significance at the 1%, 5%, and 10% levels, respectively.

**Table 4:** Series of hypothesis tests.

Null Hypothesis	F-Value
Whether the age structure variable parameters are jointly significant: *c*_1_ = *c*_2_ = *c*_2_ = *c_3_* = *c*_4_ = *c*_5_ = *c_6_* = *c_7_* = *c_8_* = *c_9_* = *e*_11_ = *e*_21_ = *e*_31_ = *e*_41_ = *e*_12_ = *e*_22_ = *e*_32_ = *e*_42_	38.60 ***
Whether the variable parameters of male age structure were jointly significant: *c*_2_ = *c_3_* = *c*_4_ = *c*_5_ = *e*_11_ = *e*_21_ = *e*_31_ = *e*_41_	52.50 ***
Whether the variable parameters of female age structure were jointly significant: *c*_6_ = *c*_7_ = *c*_8_ = *c*_9_ = *e*_12_ = *e*_22_ = *e*_32_ = *e*_42_	30.95 ***
Is there a difference in the adult equivalence scale between males and females aged 17–22: *c*_2_ = *c*_6_	132.03 ***
Is there a difference in the adult equivalence scale between males and females aged 35–40: *c_3_* = *c*_7_	101.96 ***
Is there a difference in the adult equivalence scale between males and females aged 55–60 years: *c*_4_ = *c_8_*	124.58 ***
Is there a difference in the adult equivalence scale between men and women older than 70 years: *c*_5_ = *c*_9_	39.47 ***

Note: *** indicate significance levels of 1%, 5%, and 10%, respectively.

## Data Availability

Data used during the current study are available from the corresponding author.
